# An Efficient Trio-Based Mini-Haplotyping Method for Genetic Diagnosis of Phenylketonuria 

**DOI:** 10.22074/cellj.2016.4318

**Published:** 2016-05-30

**Authors:** Saeed Talebi, Mona Entezam, Neda Mohajer, Golnaz-Ensieh Kazemi-sefat, Masoumeh Razipour, Somayeh Ahmadloo, Aria Setoodeh, Mohammad Keramatipour

**Affiliations:** 1 Department of Medical Genetics, School of Medicine, Tehran University of Medical Sciences, Tehran, Iran; 2 Department of Molecular Medicine, Faculty of Advanced Technologies in Medicine, Iran University of Medical Sciences, Tehran, Iran; 3 Department of Endocrinology, Children’s Hospital Medical Center, Tehran University of Medical Sciences, Tehran, Iran

**Keywords:** Microsatellite Repeats, Fluorescent Dyes, Capillary Electrophoresis, Diagnostic Error, Phenylalanine Hydroxylase

## Abstract

**Objective:**

The phenylalanine hydroxylase (PAH) locus has high linkage disequilibrium. Haplotypes related to this locus may thus be considered sufficiently informative for genetic diagnosis and carrier screening using multi-allelic markers. In this study, we present an efficient method for haplotype analysis of PAH locus using multiplexing dyes. In addition, we explain how to resolve the dye shift challenge in multiplex short tandem repeat (STR) genotyping.

**Materials and Methods:**

One hundred family trios were included in this descriptive study. The forward primer of a tetra-nucleotide STR and the reverse primer of a variable number tandem repeat (VNTR) were labeled with three different non-overlapping dyes 5-carboxyfluorescein (FAM), 6-carboxy-N,N,N’,N’-tetramethylrhodamine (HEX) and 6-carboxy-N,N,N’,N’-tetramethylrhodamine (TAMRA). The polymerase chain reaction (PCR) products from each family trio were multiplexed for capillary electrophoresis and results were analyzed using Peak Scanner software.

**Results:**

Multiplexing trio products decreased the cost significantly. The TAMRA labeled products had a significant predictable shift (migrated at a slower electrophoretic rate) relative to the HEX and FAM labeled products. Through our methodology we achieve, the less inter-dye shift than intra-dye shift variance. Correcting the dye shift in the labeled products, according to the reference allele size, significantly decreased the inter-dye variability (P<0.001).

**Conclusion:**

Multiplexing trio products helps to detect and resolve the dye shift accurately in each family, which otherwise would result in diagnostic error. The dye system of FAM, HEX and TAMRA is more feasible and cheaper than other dye systems.

## Introduction

The phenylalanine hydroxylase (* PAH*) gene (MIM:612349) is the well-known responsible gene for classic phenylketonuria (PKU). It is the most common inborn error of amino acid metabolism with a prevalence of approximately 1 in 10,000 births among Caucasians (1). *PAH* is located on chromosome 12q22-24.1 encompassing a 100 kb genomic region (2). The region shows high linkage disequilibrium (LD) at distances of 22 and 31 kb at either end of the gene, this in turn leads to high association of particular mutations and haplotypes. Haplotype analysis therefore becomes not only a useful approach for diagnostic purposes, but also for population genetic, demographic and epidemiologic studies (3,4). 

There are three kinds of polymorphic markers which combined together, generate extended *PAH*-locus haplotypes. These markers comprise seven diallelic restriction fragment length polymorphisms (RFLPs) scattered throughout the genomic region, a variable number tandem repeat (VNTR) at approximately 3 kb downstream of the last exon (5), and an intragenic short tandem repeat (STR) in intron 3 (6). The alternative is a mini-haplotype comprising only the STR, one RFLP (Xmn1) and the VNTR, that is informative enough and conveniently performable by PCRbased methodology (7). The best approach for unambiguously determining these haplotypes is pedigree-based analysis. 

Capillary electrophoresis (CE) technology based on automated laser induction of fluorescence polymerase chain reaction (PCR) products, has revolutionized marker genotyping and precise allele sizing. Nevertheless, the most important factors in haplotype analysis according to CE results are accurate allele calling and the expense of the procedure. Multiplexing systems are among the different approaches to reduce the cost. In the case of trio-based studies in which the affected offspring and both parents are evaluated simultaneously, two approaches can be considered. Here, we named them Individual multiplexing system, (relies on mixing all the PCR products of each individual separately) versus Trio-based multiplexing system (which relies on mixing the PCR products of trio members). Both systems are based on multiplex dye sets for labelling the overlapping fragments. There are some compatible sets of dyes which can be selected according to the guidelines of capillary instruments, local availability and the price. Each dye set requires its own instrumental spectral calibration. 

Additionally, fluorescent dyes influence the mobility of PCR fragments during electrophoresis. This may lead to misinterpretation of allele sizing in different ranges (8) and possible misdiagnosis. This discrepancy is dependent on the physicochemical properties of the fluorophore dye and the length of the fragment (9). 

By considering different approaches, in this study, we report an efficient method for familybased indirect genetic diagnosis of phenylketonuria using haplotype analysis, which is based on a multiplex dye system for STR, VNTR fragment amplification in each trio. Also, we overcome the dye shift challenge confounding accurate genotype calling. 

## Materials and Methods

### DNA samples

DNA samples of one hundred family trios consisting of Iranian PKU patients and healthy controls were included in the study. The study was a descriptive study and approved by the Research Ethics Committee of Tehran University of Medical Sciences. All subjects and their parents provided informed consent. 

### Mini-haplotype genotyping

All DNA samples were genotyped for both the intronic STR (TCTA)n and the 30 bp AT rich 3´-flanking VNTR. Three different non-overlapping dyes were selected for labelling the forward and reverse primers of the STR and VNTR fragments respectively as follows: The FAM labeled primers were used for amplification of the offspring genomic DNA and the HEX and TAMRA labeled primers were used to amplify the paternal and maternal markers respectively. The selected size standard for fragment analysis was LIZ labeled. Primer sequences were described previously (5,10). 

Amplification was performed in the ABI thermal cycler with denaturation at 95˚C for 5 minutes, followed by 37 cycles of 95˚C for 30 seconds, 60˚C for 30 seconds and 72˚C for 30 seconds, and a final extension of 72˚C for 7 minutes using Taq DNA Polymerase 2x Master Mix (Ampliqon, Denmark). PCR products were examined by electrophoresis on a 1.5% agarose gel. 

For each family trio, 5 µl of each PCR product was mixed to run one single fragment analysis reaction using the Applied Biosystems 3137 Genetic Analyzer. Capillary electrophoresis was provided by Pishgam. Biotech Company (www.pishgambc.com). Genescan results were analyzed by Peak Scanner Software v 1.0. 

### Analytical evaluation of the method

Dye shift analysis was based on evaluating two parameters: the inter-dye shift between FAM, HEX and TAMRA within each run, and the intradye shift of each dye among runs. The expected size of each allele, referred to as the "reference allele size", was taken from the human reference genome, GRCh38.p2 assembly. The accuracy of microsatellite allele sizing was confirmed in each family by comparing the results of the parents and the offspring. The validity of the sequences and core repeats was confirmed by Sanger sequencing in some families. 

The differences between the dye labeled product size (observed allele size) and the reference allele size for each allele was calculated (dye shift) and the mean size differences were then used as the basis of correction. The polynomial regression equations which relate the observed allele size to the correction coefficients were calculated (http:// www.arachnoid.com/polysolve). For correction, the "calculated correction coefficient" related to each observed allele was subtracted from the observed allele size. 

In order to determine the intra-dye shift variability we calculated the dye shift standard deviation (SD) within each dye per allelic size. Likewise, the inter-dye shift variability was analyzed between dyes for each trio per allelic size. 

For efficacy analysis the comparator method was genotyping with a single dye and multiplexing the products of each trio member separately. 

### Statistical analysis

According to normal or abnormal distributions, groups were compared by parametric or nonparametric tests. Friedman test was used to examine the significance of inter-dye variability. To determine the relationship between reference allele size and dye shift, correlation analysis was done. For statistical analysis, SPSS version 16 software was used and P<0.01 was considered significant. 

### Results

**Fig.1:**
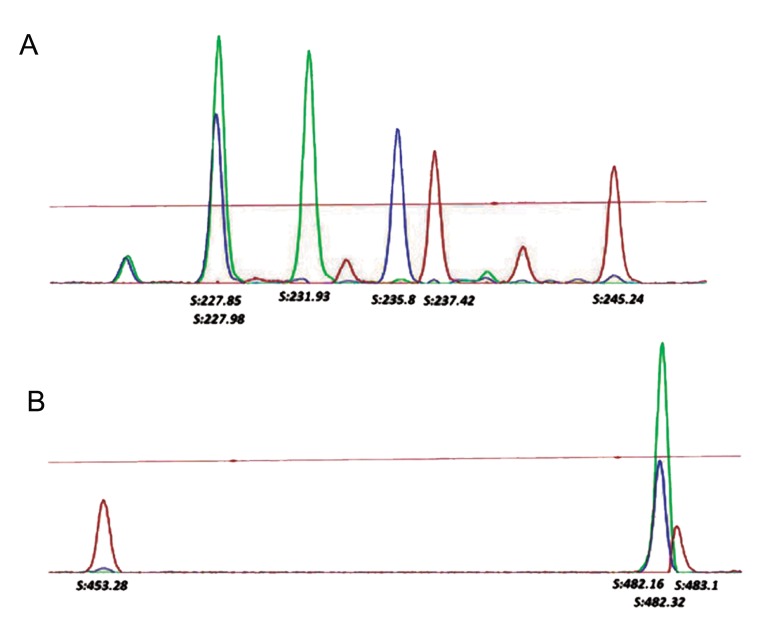
The electropherograms of PCR amplification products in a trio with a phenylketonuria patient. A. STR electropherogram and B. VNTR electropherogram. The blue (FAM), green (HEX) and red (TAMRA) peaks represented the child, father and mother respectively. The child alleles for STR (228 bp) and VNTR (482 bp) are concordant with paternal alleles. The maternal allele discrepancy in the STR marker is more than in the VNTR marker. S; Size, PCR; Polymerase chain reaction, STR; Short tandem repeat, VNTR; Variable number tandem repeat, FAM; 5-carboxyfluorescein, HEX; 6-carboxy-N,N,N’,N’-tetramethylrhodamine and TAMRA; 6-carboxy-N,N,N’,N’-tetramethylrhodamine.

Here, to determine the haplotypes of all trio members in one electrophoresis run, labeledprimers with one of three different fluorophore (FAM, HEX and TAMRA) were selected for DNA amplification of each family member. STR and VNTR amplified fragments, were labeled with the same dye set given their non-overlapping allele size range. Figure 1 represents the electropherograms of PCR amplification products in a trio. Allele sizing could be assessed unambiguously for all investigated samples. Characteristics of each marker and the corresponding allele frequencies, as well as all individual haplotypes were determined in PKU patients and healthy controls trios. These data has been used for population study of Iranian PKU families (unpublished data). 

There were some variations in allele sizing among the three dyes, but the most significant difference was observed between TAMRA when compared with both FAM and HEX. In order to validate the allele sizing and overcome the dye shift errors, the mean size of differences with respect to each reference allele was assumed as the correction coefficient for that reference allele size. The polynomial regression equations were then used to facilitate calculating the correction coefficients for the observed alleles (Table 1). According to this factor, the corrected allelic size was adjusted for all data. Table 2 shows the comparison of dye shift errors (intra-dye and inter-dye shift variability) between raw and corrected data. As specified, after correcting the capillary electrophoresis results, the inter-dye shift variability (SD) between three fluorophore-dye decreased significantly (P<0.001). Also, this variability was less than the intra-dye shifts variability (SD). 

**Fig.2:**
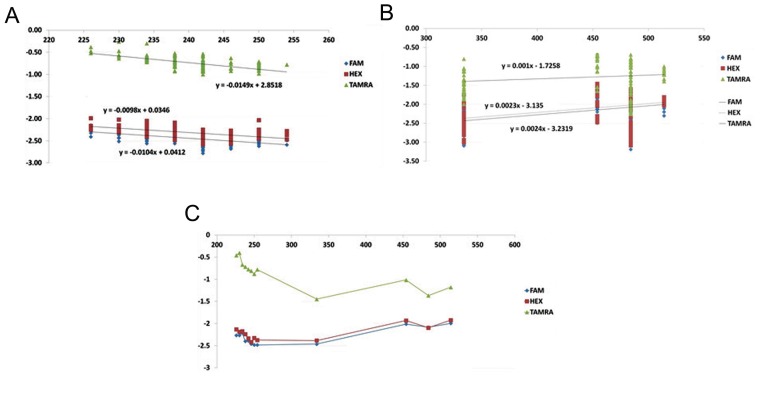
Variation of dye shift among the three dyes depending on the allelic size. The X axis indicates the reference allele size and the Y axis indicates the amount of dye shift relative to the reference allele size. TAMRA showed the smallest difference. A. Negative correlation between the reference allele size and the dye shift in three dye-labeled products less than 300 bp, B. Positive correlation in VNTR products between 300 to 600 bp and C. Decreased variation of three labeled products with increase in fragment size. The difference in the slope of the curves between less than 300 and more than 300 on the X-axis indicating the influence of the fragment size on the dye shift error. As illustrated TAMRA fragments showed the less difference with the reference allele size versus FAM and HEX. FAM; 5-carboxyfluorescein, HEX; 6-carboxy-N,N,N’,N’-tetramethylrhodamine, TAMRA; 6-carboxy-N,N,N’,N’-tetramethylrhodamine, F; FAM, H; HEX and T; TAMRA.

Furthermore, our data represented a significant correlation between the dye shift and the allelic size before correction (P<0.001) which resolved after shift correction (P>0.05). In tetra-nucleotide STR repeats with less than 300 bp length there was a significant negative correlation between reference allele size and the dye shift in all dye labeled products (rFAM=-0.565, rHEX=-0.552, rTAMRA=-0.610, P<0.001) but in thirty-nucleotide VNTR repeats with size ranging from 300 to 600 bp, there was a positive correlation which was significant only in FAM and HEX labeled products (Fig.2A,B). Moreover, the mean allelic sizes estimated with all labeled fragments were smaller than the reference allele size. The smallest deviation belonged to TAMRA. Also, variation in allele sizing among the three dyes decreased more dramatically as allele size increased in fragments less than 300 bp compared with larger fragments (Fig.2C). Comparative characteristics of two multiplexing strategies discussed in this paper, is summarized in Table 3. 

**Table 1:** The correction coefficients and polynomial regression equations


Reference allele size (R)	Correction coefficient		
	FAM-R	HEX-R	TAMRA-R

226	-2.26	-2.13	-0.46
230	-2.34	-2.19	-0.52
234	-2.35	-2.23	-0.63
238	-2.4	-2.27	-0.72
242	-2.52	-2.38	-0.81
246	-2.52	-2.42	-0.81
250	-2.48	-2.33	-0.81
254	-2.48	-2.37	-0.78
334	-2.46	-2.38	-1.43
454	-1.91	-1.9	-0.98
484	-2.16	-2.1	-1.37
514	-2.07	-1.92	-1.18


The polynomial regression equations (based on the data above) for each dye (x indicates the “observed allele size” and f(x) represents the “calculated correction coefficient”): FAM equation (R^2^=0.98, SE=0.028): f(x)=5.87e+2+-1.06e+1x+7.79e-2x^2^+-3.02e-4x^3^+6.47e-7x^4^+-7.28e-10x^5^+3.36e-13x^6^ HEX equation (R^2^=0.97, SE=0.031): f(x)=5.62e+2+-1.01e+1x+7.44e-2x^2^+-2.88e-4x^3^+6.16e-7x^4^+-6.93e-10x^5^+3.20e-13x^6^ TAMRA equation (R^2^=0.99, SE=0.036): f(x)=1.30e+3+-2.33e+1x+1.71e-1x^2^+-6.62e-4x^3^+1.41e-6x^4^+-1.57e-9x^5^+7.20e-13x^6^ FAM; 5-carboxyfluorescein, HEX; 6-carboxy-N,N,N’,N’-tetramethylrhodamine, TAMRA; 6-carboxy- N,N,N’,N’-tetramethylrhodamine and R^2^; The coefficient of determination.

**Table 2:** Dye shift comparison. As illustrated, the inter-dye shifts are less than the minimum of intra-dye shifts in each respective allelic size range


Reference allele size (R)	Intra dye shift variability (SD)			Inter dye shift variability (SD)	
	FAM	HEX	TAMRA	Before correction All three dyes	After correction All three dyes

226	0.103	0.132	0.070	0.76	0.05
230	0.108	0.097	0.126	0.78	0.04
234	0.089	0.089	0.091	0.72	0.04
238	0.093	0.102	0.115	0.80	0.04
242	0.104	0.078	0.115	0.89	0.04
246	0.101	0.089	0.097	0.64	0.02
250	0.073	0.104	0.080	0.77	0.05
254	0.110	0.095	N	0.35	0.02
334	0.319	0.308	0.272	0.49	0.08
454	0.322	0.349	0.34	0.45	0.07
484	0.431	0.480	0.489	0.44	0.11
514	0.122	0.077	0.125	0.41	0.03


N; Not-determined.

**Table 3:** Comparison of two alternative multiplexing strategies


Multiplexing strategies	Advantage	Disadvantage

Individual multiplexing system	Lower dye shift variation	More electrophoretic interrun error
		More analyzing time
		More analyzing cost per run
Trio-based multiplexing system	Lower electrophoretic inter-run variation	More dye shift variation (correctable)
	Longer analysis	
	Higher cost per run	


## Discussion

Here we suggest a more cost-effective and feasible multiplex dye set for multi-allelic marker genotyping in trios studies based on FAM, HEX and TAMRA fluorophore labelling and fragment analysis. Using this dye set in a pedigree-based study, we demonstrated a size-dependent dye shift which evidently can be resolved.

In addition to determining the origin of mutations and allele distributions in different populations, PAH mini-haplotyping is an informative indirect method for genetic diagnosis and carrier screening of PKU patients. It will be applicable particularly in families with one affected individual referred for genetic analysis and facilitates pre-implantation genetic diagnosis and prenatal diagnosis.

Capillary electrophoresis is the gold standard method in marker genotyping, nevertheless, there are some sources of errors that needs to be minimized, such as, sampling errors, genotyping errors, including allelic dropout and null alleles (11, 12) along with PCR (13) or electrophoresis artifacts (14) and errors in allele size calling, which, one reason can be dye shift (15).

Furthermore, reducing the overall cost is another consideration. This would be achieved by different multiplexing systems based on PCR multiplexing or post-PCR multiplexing -also named pseudo-multiplexing or poolplexing (16). Further, the latter could be categorized into two distinct strategies in regards to trios studies. Simple individual multiplexing system which relies on mixing all the PCR products of each individual separately and an alternative trio-based multiplexing system, represented in this study. In each of the two approaches, a PCR-multiplexing step can also be integrated as well.

The trio-based multiplexing system, based on using a distinctive fluorophore primer for each member of the trio, enables simultaneous haplotype analysis. Such trio multiplexing can effectively increases the accuracy of analysis while reducing time. We used polynomial regression equations based on our data to predict correction coefficients which can be used in combination with other allele sizing softwares. This inevitably facilitates allele sizing and increases accuracy (Table 3).

Despite the fact that ordering three different labeledprimers for each locus seems to increase the cost, reducing the cost per run for each family decreases the overall price dramatically, and subsequently makes it a cost-effective approach, particularly for centers which offer PKU genetic testing and prenatal diagnosis (PND) or preimplantation genetic diagnosis (PGD) services.

Also, trio multiplexing results in detection and resolving the dye shift error in each family. Correcting the labeled fragments allelic size using correction coefficients can significantly decrease the inter-dye shift. There are limited number of studies which explore the FAM, HEX and TAMRA dye set mobility shifts (9, 17, 18). According to previous studies, when using rhodamin dyes such as TAMRA and ROX (6-carboxy-X-rhodamine), penta-nucleotide-repeat fragments show 4.75 b shift from fluorescein dye fragments such as FAM, HEX, JOE (6-carboxy-2’,7’-dimeoxy-4’,5’dichlorofluorescein) and NED. The degree of retardation decreases with increasing size, regardless of the fluorophore label (9). Haberl and Tautz (17) reported a slight dye mobility shift of 0.7 b among TAMRA and FAM and smaller retardation between HEX and the two other dyes. Also, a 2.07 bp to 3.68 bp size range difference was represented when four different FAM, NED, PET and VIC dyes were applied to a single fragment (18). Evaluating the dye shift error in this study is based on statistical analysis (P<0.001), which is superior to previous analyses based only on SD comparisons. The observed TAMRA retardation in this study decreased with increasing size (as expected) which is predominant in fragments less than 300 bp; although the sequence of the repeat core may influence this shift.

More detailed investigations showed that the structure of the attached dye influences the effective charge of the fragment and its molecular migration through the capillary media. Dyes with more negative charges migrate faster. However, the relative fluorescent dye charge is the predominant factor in determining DNA mobility, even though it can be influenced by dye-DNA base interactions (8).

With respect to capillary electrophoresis instruments, different specified dye sets can be selected for primer labelling. Here we showed that the (FAM, HEX, TAMRA) set can be used appropriately in replace of (FAM, HEX and NED) usual dye set in combination with the LIZ size standard. Due to the similarity of spectral characteristics of dyes, the more cost-effective TAMRA dye, can be an alternative for NED while also being compatible with the same matrix standard used for spectral calibration of the instrument (DNA Fragment Analysis by Capillary Electrophoresis, ABI, user guide). 

Combination of length, sequence and physicochemical properties of the fluorophore label, determine the migration behavior of each fragment in denaturing CE. So in the case of similar length and sequence, in 100 family trios we can determine the TAMRA labeled products discrepancy in comparison with FAM and HEX ones, and overcome this dye shift error, which can disturb allele sizing specifically in di-nucleotide STR genotyping and leads to diagnostic error. 

It is worth to mention that identification of all genotyping errors and the nature of the errors might not be possible all the times (19). 

## Conclusion

In this study, we show that trio-based analysis in a single capillary electrophoretic run may decrease the testing costs and significantly facilitate the analysis when using the method in a diagnostic referral laboratory. As well as reducing the cost, we also demonstrate that the trio-based multiplexing system, after correction , leads to more accurate haplotype analysis. Eventually, all individual haplotypes were determined according to the corrected data for further investigations. 

Furthermore, the suggested correction coefficients for each allelic size could be considered as an adjustment for genotype calls of every repeat length polymorphic marker with capillary electrophoresis. 
